# Access to Hysterectomy—What Is the Realistic Rate for Pure Vaginal Hysterectomy? A Single-Center Prospective Observational Study

**DOI:** 10.3390/jcm13206130

**Published:** 2024-10-15

**Authors:** Felix Neis, Aylin Ayguen, Romina-Marina Sima, Erich-Franz Solomayer, Ingolf Juhasz-Boess, Gudrun Wagenpfeil, Percy Brandner, Klaus Joachim Neis

**Affiliations:** 1 Department of Obstetrics and Gynecology, University Hospital Tübingen, 72074 Tübingen, Germany; 2 Department of Obstetrics and Gynecology, University Hospital Homburg, 66421 Homburg, Germany; 3 Department of Obstetrics and Gynaecology, University of Medicine and Pharmacy “Carol Davila”, “Bucur” Maternity, 020956 Bucharest, Romania; 4 Department of Obstetrics and Gynecology, University Hospital Freiburg, 79106 Freiburg, Germany; 5 Institute of Medical Biometry, Epidemiology and Medical Informatics, Saarland University, 66421 Homburg, Germany; 6 Frauenärzte Saarbrücken West, 66113 Saarbrücken, Germany; 7 Frauenärzte am Staden, 66121 Saarbrücken, Germany; kjneis@gyn-saar.de

**Keywords:** access to hysterectomy, minimally invasive hysterectomy, reduction in abdominal hysterectomy, vaginal hysterectomy

## Abstract

**Background/Objectives:** Hysterectomy (HE) is the most common surgical procedure in gynecology worldwide. The guidelines of most countries unanimously recommend vaginal hysterectomy (VH) as the access of first choice. However, there are significant international differences in the implementation of this recommendation. **Methods:** In the consistent implementation of the national guidelines, the aim of this prospective observational cohort study was to evaluate how many hysterectomies can be performed vaginally under real-world conditions for benign indications excluding genital prolapse and extensive endometriosis. For this purpose, the requirements of the guidelines were implemented for all HE cases. All HEs were performed by a single, experienced surgeon. The aim was not to go to the limits of the method, but to develop a reproducible benchmark with the lowest possible complication rate. **Results:** From 2011 to 2020, 230 hysterectomies were performed for benign indications. A VH was performed in 146 cases (63.5%), and a laparoscopic hysterectomy (LH) in 75 cases (32.6%). An abdominal hysterectomy (AH) was only required in nine cases (3.9%). The decision for LH was made in half of the cases due to the assumed presence of endometriosis or a significantly enlarged uterus. The median duration of VH was 32 min (range 16–118 min), and the uterine weights were 15–540 g. The rate of postoperative complications of VH was 3.4%. **Conclusions:** In line with international guidelines, VH is possible in over 60% of cases with a short surgical time and a low complication rate. LH procedures are useful in the presence of assumed additional pathology in 35%. AH is reserved for huge uteri. A reduction in AH below 10% is possible. The global target could be a rate of 60–30–10% for VH, LH, and AH.

## 1. Introduction

Hysterectomy (HE) is a surgical procedure that helps effectively and permanently relieve symptoms originating in the uterus, such as hypermenorrhea, dysmenorrhea, or dyspareunia, which can detrimentally affect patients’ quality of life [1,2].

Before 1989, only two surgical procedures were available: vaginal hysterectomy (VH) and abdominal hysterectomy (AH), accounting for 70% and 30% of cases, respectively [3].

In 1989, Reich first performed a total laparoscopic hysterectomy (TLH) [4]. Subsequently, the list of procedures was expanded to include laparoscopically assisted vaginal hysterectomy (LAVH) and laparoscopically assisted supracervical hysterectomy (LASH) [4,5]. These minimally invasive methods aim to reduce the number of AHs performed. However, in several cases, VH has been replaced by laparoscopic procedures, despite guidelines commonly recommending VH as the first-choice approach [2,6]. The ongoing departure from VH has resulted in the required technical skills being lost and even excluded from surgical training in many parts of the world. However, efforts to re-introduce this surgical procedure are now being made in Europe and the United States [7,8,9]. Since the first FDA warning on the use of power morcellators, there has been a decline in laparoscopic supracervical hysterectomy [10]. These procedures have also been mainly replaced by laparoscopic procedures [11,12,13,14]. VH does not tend to have any influence here. A recent retrospective analysis of vaginal hysterectomies in the USA showed an ongoing decreasing utilization of VHs [11,15].

With the introduction of LAVHs in 1991, we succeeded in reducing the AH cases to 10% of all performed HEs within a short period, applying the two minimally invasive methods, VH and LH, to 60% and 20% of cases at that time [5]. This study aimed to evaluate the proportion of HE types (VH, LH, and AH) among patients indicated for surgery, provided the relevant guidelines were strictly adhered to.

## 2. Materials and Methods

**Figure 1:**
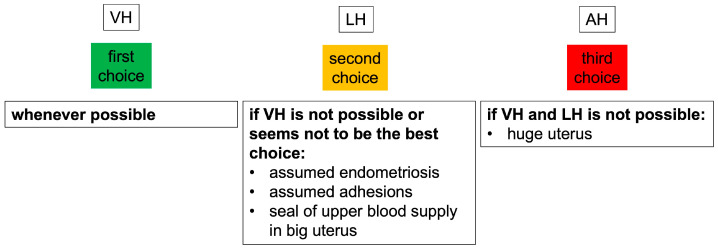
Choosing the hysterectomy approach. Cases of prolapse and deep-infiltrating endometriosis were excluded. VH = vaginal hysterectomy, LH = laparoscopic hysterectomy, AH = abdominal hysterectomy.

To define a realistic rate of VHs under real-world conditions, a surgeon (Klaus Joachim Neis—KJN), experienced in HEs for all indications (endometriosis, prolapse, and oncology), consistently followed the guidelines in terms of indication and access route for all HEs performed. Only patients with HEs for benign indications were included in this study. Patients with oncological indications, prolapse, or surgery for suspected deep-infiltrating endometriosis, with foreseeable requirements for additional complex procedures, were excluded, as the possible routes of pure hysterectomy without additional procedures were evaluated in this study. VH should be performed whenever possible. If VH is not possible, the second choice should be LH, while AH should only be used if neither VH nor LH are possible. While laparoscopic surgery should not be performed if vaginal access is possible (Figure 1), LH is no longer limited to LAVH. All available types of HE (LAVH, LASH, and TLH) were used.

Data on variables such as indications, route of access, need for morcellation, duration of surgery, patient age, intra- and postoperative complications, histological results, and uterine weight, were collected prospectively and entered into a Microsoft Access table (Microsoft Corporation, 2010, Redmond, WA, USA).

This observational study took place over a period of 10 years, capturing 250 expected HEs. There was no interim analysis. All surgeries were performed at the Department of Obstetrics and Gynecology, University Hospital Homburg, Germany.

The indications for HE were determined by a surgeon (KJN). The surgeon examined the patient, performed a transvaginal ultrasound, and discussed the indications for HE with the patient, including possible access routes and alternatives applicable. The formerly classic contraindications such as multiple cesarean sections, a “negative traction attempt”, or narrow vagina, played no or only a subordinate role in the choice of access route. In the group of patients in whom simultaneous endometriosis could not be excluded based on their medical history or after preoperative vaginal examination with transvaginal ultrasound, LH was always performed even if VH was possible. AH was performed in cases of large uteri protruding beyond the umbilicus, where VH and LH would cause possible difficulties. This information was provided in accordance with the principles of shared decision-making. The patients were included in this study by the surgeon Klaus Joachim Neis and gave their informed consent at the time of indicating the surgery.

VH was performed according to the classic technique using the BiClamp^®^ (ERBE, Tuebingen, Germany) [16,17].

After 1–3 weeks postoperatively, all patients presented to the surgeon for a final examination and discussion of histology. This process ensured that complications that occurred after discharge were recorded.

This study was approved by the Ethics Committees of the Medical Faculty of the Saarland University at the Saarland Ärztekammer, Germany (Number: Bu 200/21, 31 January 2022). All procedures were performed in accordance with the ethical standards of our institutional research committee. All patients provided informed consent before surgery.

The statistical analysis was performed by the Institute of Medical Biometry, Epidemiology and Medical Informatics, Saarland University, Homburg/Saar, Germany.

Statistical analysis was performed with Microsoft Excel (Version 16.84, Microsoft Corporation, 2024, Redmond, WA, USA) and IBM SPSS 27.0 (IBM Corp. Released 2020. IBM SPSS Statistics for Windows, Version 27.0. Armonk, NY, USA: IBM Corp).

Quantitative data were examined for normal distribution using the Shapiro–Wilk test. Most variables did not follow a normal distribution. Thus, we used the Mann–Whitney U test for comparisons between two groups and the Kruskal–Wallis test for comparisons between three groups. The *p*-values for additional two-group comparisons following the Kruskal–Wallis test were calculated with the Bonferroni correction. Two-sided *p*-values of <0.05 were considered statistically significant.

## 3. Results

**Figure 2:**
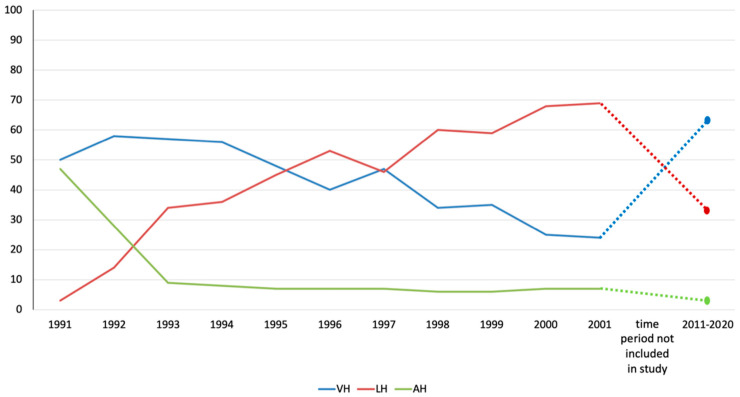
Percentage changes in the access route to hysterectomy after the introduction of LAVH (1991–2001) and during the study period (2011–2020). The time between 2001–2011 is not part of this analysis (dotted lines).

In 1991, we included LAVH on our list of HE procedures and we succeeded in reducing the rate of AH to less than 10% within 2 years. Figure 2 shows changes in the forms of HEs performed from 1991 to 2001, as well as a summary of the procedures performed in the current study period (2011–2020). Between 1991 and 2001, 2340 HEs were performed. Unfortunately, only the number of procedures and the year are known from this period. At that time, the surgical team consisted of numerous experienced and trainee surgeons under the supervision of the last author (KJN). The procedures recorded during 2011–2020, and evaluated in this study, were performed by only one surgeon (KJN) to help ensure the homogeneity of surgical indications and performance.

The increasing proportion of LHs led to a steep decrease in AHs from 47% to 9%. From 1994 onwards, in addition to AH being replaced by LH, the rate of VH decreased significantly. There was an unintentional reduction in VHs from 56% (1994) to 24% (2001) within 7 years.

Between 2011 and 2020, 230 HE procedures were performed for patients with benign uterine diseases. All procedures were performed under general anesthesia. Each patient received a single antibiotic and postoperative thromboprophylaxis.

VH was performed in 146 (63.5%) patients. LH was performed in 75 (32.6%) patients. The LH approach was used for 56 (24.3%), 15 (5.2%), and 4 (1.7%) patients undergoing LAVH, LASH, and TLH, respectively. AH was necessary in nine (3.9%) patients.

The indications for HE according to access route are shown in Table 1. In cases of multiple indications, only the leading indication was recorded. Fibroids were the main indication for HE in all groups (VH, LH, and AH) and the only indication for AH.

In patients in whom simultaneous endometriosis could not be excluded preoperatively, LH was always performed, even if surgery could have been performed purely vaginally. In 36 (48.0%) patients in the LH group, endometriosis was present, mostly exclusively peritoneal, and they were treated with the same surgery.

In the AH group, all nine women had a large uterus due to fibroids, protruding beyond the umbilicus; in a case with a rapidly growing uterus myomatosus in the postmenopausal period, a sarcoma could not be ruled out preoperatively. The final histological examination revealed benign findings.

Patient age ranged from 30 to 83 years. The mean ages for patients undergoing VH, LH, and AH were 47.8 ± 8.1 (range: 32–83), 44.4 ± 5.9 (range: 30–56), and 51.4 ± 8.0 (range: 44–64) years, respectively. There was a significant difference in age between the LH and VH groups (*p* = 0.032).

**Figure 3:**
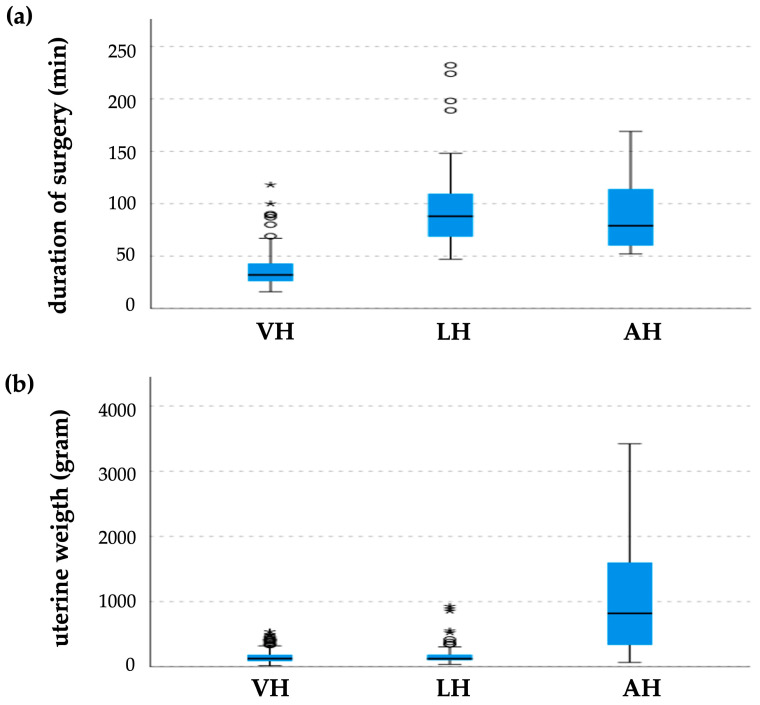
Whisker box plot: (**a**) surgery time for different types of hysterectomies; (**b**) uterine weight of different types of hysterectomies. * Extreme values; ° outlier.

The duration of surgeries performed differed significantly among the groups (Figure 3a). While the mean duration of VH was 37.1 ± 16.7 min (range 16–118 min), that of LH was 93.7 ± 38.1 min (range 47–232 min). When LH was indicated, additional interventions (such as endometriosis and adhesions) were suspected preoperatively. The duration of AH, given the exclusive indication of large fibroids, was on average 96.6 ± 45.0 min (range 52–169 min). LHs and AHs took approximately 2.5 times longer than VHs (*p* < 0.001).

The weights of the extirpated uteri were comparable in the VH (151.8 ± 104.7 g) and LH (183.3 ± 177.0 g) groups (Figure 3b). The maximum weight of the VH group was 540 g and that of the LH group was 930 g. A significant difference (*p* < 0.001) in the VH and LH groups compared to the AH group (1215.6 ± 1192.1 g) was observed, with a median weight of 820 g and a maximum weight of 3421 g.

Intraperitoneal morcellation was performed during LH in patients with LASH and TLH with uteri that could not be retrieved vaginally. In accordance with the recommendations of the German Working Group for Endoscopy and Society for Gynecology and Obstetrics [18], in-bag morcellation was not performed. In VH, morcellation is performed intravaginally or at the introitus.

Morcellation was performed in 47.9% of the VH cases (Table 2); depending on the size of the uterus and the anatomical conditions of each case, only a hemiotomy or a more complex morcellation with several steps was necessary.

The surgery time for VH without morcellation was 32.0 ± 13.5 min (range 16–118 min); the corresponding values for that with morcellation were 42.6 ± 17.7 min (range 21–100 min). Therefore, on average, a procedure with morcellation took 10.6 min (*p* < 0.001) longer than that without morcellation.

Uteri without morcellation were extirpated at 91.9 ± 49.0 g (range 15–393 g), and those with morcellation at 216.7 ± 110.4 g (range 67–540 g) (*p* < 0.001).

Simultaneous adnexal surgeries were performed in 14.8% of all HEs. In cases of VH, adnexal surgeries were performed in 7.5% (11 of 146), in cases of LH in 29.3% (22 of 75), and in cases of AH in 11.1% (1 of 9).

In total, 10 (4.3%) complications occurred intra-and postoperatively in 230 hysterectomies. All intraoperative and major postoperative complications (Clavien–Dindo III–V) were evaluated. The complications categorized by access route are shown in Table 3.

As all patients presented to the surgeon at 1–3 weeks postoperatively for a final examination with a histological evaluation, all complications were recorded.

The complication rate was 3.4% (5/146) in the VH group. These included a bladder injury recognized intraoperatively, which was treated vaginally in the same session, and a postoperative hemorrhage after approximately 12 h, which necessitated a laparoscopic revision. The remaining complications occurred 1–3 weeks postoperatively and required surgical revision under general anesthesia in two cases. In a single case, unilateral urinary kidney retention was controlled by a temporary insertion of a double-J catheter.

The complication rate of LH was 2.7% (2/75). In a single case, increased intraoperative bleeding required postoperative administration of two erythrocyte concentrates, and a revision of the vaginal cuff was necessary due to a hematoma at 2 weeks postoperatively.

A total of three cases of complications occurred in the AH group (33.3%). In a single case, the administration of two erythrocyte concentrates was necessary to control the hemorrhage intraoperatively. In another case, a lesion in the colon due to extensive adhesions was treated intraoperatively. Due to peritonitis, revision was necessary 2 days later in this case.

Conversion from VH or LH to AH was not necessary in any case.

## 4. Discussion

Using minimally invasive HE methods (VH and LH), the rate of AH can be reduced to less than 5% under real-world conditions. Applying the VH with the BiClamp^®^ technique, large uteri can be removed in a short surgery time, following the guidelines, and resulting in a low complication rate. The uptake of VH can exceed 60% for benign indications, excluding prolapse and extensive endometriosis.

Many studies have described the effects of reducing AH rates by introducing the LH methods [12,19,20]. The analysis of our data from 1991 to 2001 shows that the introduction of LH techniques reduced the AH rate from 47% to less than 10% within 2 years [5]. Alongside a decrease in the number of AHs, the number of LHs decreased by >50%, and some cases previously eligible for VH were managed with LHs. This resulted in a drastic reduction in VHs to 24% of all HEs. This effect has been described previously [11,12,15,21,22], despite all national and international guidelines favoring VH as the first-choice method [1,2,9,23]. Despite these recommendations, the preferred approach to hysterectomy in most European countries [12,22,24] and the USA [11,15] is a laparoscopic approach. In the USA [11,15], the second most common approach, at approximately 19%, is AH. VHs are only performed in 13–15.3% of cases. In some regions of Germany, similar trends can be seen, but with balanced rates of VHs and AHs [24]. However, the rate of AHs and VHs also appears to be decreasing here in favor of LHs. Data on regions in which VH is the first access route to HE are available from Germany [5,25], Austria [19], and Finland [26]. All these publications show that a reduction in AHs can be achieved with the help of minimally invasive forms of hysterectomy (VH and LH).

Our study shows that prioritizing VH remains a valid approach for 75.3% of the cases. However, because we decided to perform LH in cases of suspected associated endometriosis, the VH rate decreased to 63.5%. It is reasonable to replace VH with LH in these cases, as demonstrated by the fact that we found intraperitoneal endometriosis in 48% of the cases, which was then treated laparoscopically. In 20.8% of cases, AH could be replaced by LH, as desired, mostly due to the large uteri. This resulted in 4.6% of cases which still require AH. This evidence corresponds to the findings from our previous study, in which the AH rate was reduced to 7–8% after the implementation of LAVH [5].

The surgery time for VH using the BiClamp^®^ technique is approximately 2.5 times shorter than for LH and AH, as previously shown [27]. A retrospective database analysis of more than 161,000 patients in the USA showed an increase in operative time for VH and a decrease for LH, despite VH still maintaining the shortest operative times [11]. As the overall rate of VH decreased from 19.3% in 2017 to 15.3% in 2020 in the USA, a loss of operational capability due to the reduced utilization of VH may be the reason for the increase in surgery time. The shorter surgical time increased surgical capacity for other procedures. The low variation in surgery time the indicates excellent reproducibility of VH using this technique. A further advantage of VH is the use of only a few instruments, which require low maintenance, making it more cost-effective than LH and AH [8,28,29,30].

VH can be performed on uteri weighing up to 500 g; however, techniques for vaginal morcellation must also be mastered. The surgical time was extended by an average of 10 min if vaginal morcellation was necessary. Similar changes in surgical time have been reported [31,32]. The risk of cell spilling in vaginal morcellation exists in principle, but evidence on the observed spillage is limited [33], and thus neither German nor American guidelines include any recommendations on vaginal morcellation [2,34].

In this study, AH was indicated when the fundus protruded beyond the umbilicus. Although it is also possible to manipulate larger uteri minimally invasively, vaginally, or laparoscopically [35], the purpose of our observational study was not to explore the limits of the individual procedures but to find the most effective, safest, and most sustainable solution for the patient in each individual case. Therefore, conversion was not necessary in any of the cases. Similarly, no blood transfusion was required for any patient undergoing a VH. The VH complication rate of 3.4%, which is consistent with that reported in previous studies [11,26,27,36], reflects the correct selection of patients and good reproducibility.

This study had some limitations. The HEs were performed by an experienced surgeon and the limits of the methods were not tested in either the VH or LH approaches. The number of 230 HEs provides a good basis for analyzing the possible rate of VH under real world conditions. A better statement, especially for the comparison of complication rates, would be possible with a higher number of HEs. A multicenter study with more surgeons could also lead to a more precise conclusion. However, as all surgeries were performed by a single surgeon, a baseline can be established without deviations in respect of patient selection and the performance of surgery, which can be confirmed by further studies. The feasibility of the observed results needs to be proven by less experienced surgeons or in training settings.

Notably, vaginal–adnexal procedures were rarely performed in our study cohort. However, there is sufficient evidence to suggest that both vaginal salpingectomy and adnexectomy can be safely performed [3]. This is in line with some recommendations that adnexal interventions are not contraindicated when planning VH [37].

## 5. Conclusions

In conclusion, this prospective observational study demonstrated the advantages of VH as the first-choice method. VH was possible in two-thirds of cases without stretching the limits of the method. LH is the method of choice in the presence of additional pathologies, particularly occult intraperitoneal endometriosis. AH was reserved for large uteri.

The advantages of VH include short surgery time, low complication rate, and comparatively low costs. The global targets could be 60%, 30%, and 10% for VHs, LHs, and AHs, respectively.

## Figures and Tables

**Table 1:** Preoperative indications for hysterectomy according to access route and after the exclusion of known postoperative endometriosis.

Indications								
VH			LH			AH		
Indication	N	Percent	Indication	N	Percent	Indication	N	Percent
Fibroids	68	46.6%	Fibroids	37	49.3%	Fibroids	8	88.9%
Bleeding disorders	36	24.7%	Endometriosis	36	48.0%	Fast-growing fibroids	1	11.1%
CIN3/AIS	24	16.4%	Lynch syndrome	2	2.7%			
Dysmenorrhea	16	11.0%						
Atypical hyperplasia of the endometrium	2	1.3%						
Total	146	63.5%		75	32.6%		9	3.8%
Total (after exclusion of all endometriosis cases)	146	75.3%		39	20.1%		9	4.6%

**Table 2:** Morcellation in vaginal hysterectomy. Comparison of surgical times and uterine weights of cases of VH with and without morcellation.

Morcellation	With Morcellation	Without Morcellation	*p*-Value
N (%)	70 (47.9%)	76 (52.1%)	
Surgery time (min)	42.6 ± 17.7	32.0 ± 13.5	*p* < 0.001
Range (min)	21–100	16–118	
Weight (g)	216.7 ± 110.4	91.9 ± 49.0	*p* < 0.001
Range (g)	67–540	15–393	

**Table 3:** Intraoperative and postoperative complications by access route. * Same patient.

	VH	LH	AH
N (%)	5/146 (3.4%)	2/75 (2.7%)	3/9 (33.3%)
Intraoperative complications			
	1× Bladder injury	1× Bleeding	1× Bleeding
			1× Injury to the colon *
Postoperative complications (Clavien-Dindo III–V)			
	1× Hematoma in the pouch of Douglas (after 12 h)	1× Hematoma at the vaginal cuff (after 2 weeks)	1× Peritonitis after primary suture of the colon due to injury during extensive adhesiolysis (after 3 days) *
	1× Pain at the vaginal cuff (after 7 days)		
	1× Urinary retention (after 7 days)		
	1× Vesicovaginal fistula (after 3 weeks)		

## Data Availability

The original contributions presented in this study are included in the article; further inquiries can be directed to the corresponding author/s.
